# Information Needs of Patients with Inflammatory Bowel Disease in the Digital Era: A 20-Year Longitudinal Study

**DOI:** 10.3390/jcm14113939

**Published:** 2025-06-03

**Authors:** Alberta L. A. Ajani, Derk Frank, Andreas Raedler, Martina E. Spehlmann

**Affiliations:** 1 Department of Internal Medicine III, University of Schleswig-Holstein, Arnold-Heller-Straße 3, 24105 Kiel, Germany; 2 Department of Internal Medicine and Gastroenterology, Asklepios Westklinikum Hamburg, Suurheid 20, 22559 Hamburg, Germany

**Keywords:** inflammatory bowel diseases, information deficits, e-health, counselling, online consultations, biologics, therapy

## Abstract

**Background:** Chronic inflammatory bowel disease significantly impacts patients’ everyday lives. Despite receiving regular medical care in gastroenterological or family medicine consultations, patients with inflammatory bowel disease (IBD) still experience a lack of information. To evaluate these deficits, we analyzed the main points of interest raised in an online consultation forum offered as a supplementary resource for patients. **Methods**: We analyzed 20 years of online consultation data at three time points, 2003 (launch of the forum), 2013, and 2024, and compared them against each other. A total of 681 patients participated in the consultations during these years. The clinical profiles of the participants included Crohn’s disease (CD, n = 209), ulcerative colitis (UC, n = 140), unclassified colitis (IBDU, n = 30), and individuals with no specified diagnosis (NSD, n = 303). **Results**: Patients with ulcerative colitis demonstrated interest in topics such as diet and nutrition, as well as treatment with biologics. Patients with Crohn’s disease expressed interest in diet, nutritional management, and treatment with biologics. Additionally, they showed interest in pain management, diagnostic imaging, and stress management. In the case of patients with unclassified colitis, a broad range of topics was addressed, with no single area emerging as particularly prominent. Patients with no specified diagnosis exhibited interest in diet and nutrition, laboratory diagnostics, and pain therapy. **Conclusions**: For patients with inflammatory bowel disease, online consultations represent a valuable complement to standard medical care. They provide additional support and contribute to enhancing patients’ confidence in managing their condition. A broad spectrum of disease-related topics was addressed during the consultations. Identified information gaps can be systematically discussed and subsequently reduced.

## 1. Introduction

In Germany, the average duration of a medical consultation, whether outpatient or inpatient, is approximately five minutes [1]. Within this limited timeframe, clinicians are expected to assess symptoms, establish a diagnosis, and discuss potential treatment options. However, empirical studies indicate that patients remember only about 10% of the information conveyed during these encounters. Consequently, misunderstandings and unanswered questions after consultation are common [2]. Patient advocacy groups have repeatedly pointed out significant information deficits following medical appointments. These gaps in understanding may negatively impact treatment adherence, disease management, and health outcomes, particularly in patients with chronic conditions who require frequent and detailed consultations. The integration of secure digital communication platforms presents a valuable complement to traditional consultations. By enabling asynchronous interaction outside regular office hours, these tools offer patients the opportunity to clarify medical information in a less time-pressured, more reflective setting. This format also encourages the discussion of topics that might be perceived as sensitive or challenging during brief in-person encounters.

For physicians, digital follow-up can provide deeper insights into patient concerns, highlight frequently misunderstood issues, and support more personalized care strategies. As healthcare systems continue to evolve, structured digital communication should be considered a meaningful supplement to conventional consultations, with the potential to improve patient understanding, satisfaction, and clinical outcomes [3,4]. Inflammatory bowel disease (IBD), classified as a chronic condition, encompasses Crohn’s disease (CD) and ulcerative colitis (UC) as its two most prevalent forms. Approximately 10–15% of people with inflammatory bowel disease cannot be clearly classified as having either Crohn’s disease or ulcerative colitis; these cases are referred to as unclassified colitis (IBDU) [5]. The disease typically usually manifests in young adulthood, although it can occur at any age, including early childhood. Crohn’s disease can occur in any part of the digestive tract; however, the terminal ileum is most commonly affected. Symptoms include abdominal pain, diarrhea, weight loss, malabsorption, and malnutrition. The inflammatory process in the intestine is localized, segmental, and transmural. Additionally, the development of fissures, fistulas, abscesses, and intestinal obstructions is possible [6]. In ulcerative colitis, the entire colon can be affected. Symptoms include diarrhea, abdominal pain, and weight loss. The primary goals of treatment are to relieve symptoms, reduce inflammation, and achieve long-lasting remission. Therapy also aims to prevent potential complications. The choice of medication depends on the severity of disease activity, which is classified as mild, moderate, or severe. In recent years, many new drugs have been approved for inflammatory bowel disease treatment. A wide range of medications is now available for treating acute flare-ups and for both the induction and maintenance of remission. In clinical practice, treatment commonly includes corticosteroids, immunomodulators, biologics, integrin inhibitors, IL-12/23 or IL-23 blockers, 5-aminosalicylates, antibiotics, and other supportive therapies [7,8]. The initiation of drug therapy should be guided by the individual course of the disease and the patient’s known risk profile. In severe cases that cannot be controlled with medication, or if complications arise, surgery may be necessary [9,10]. Unclassified colitis is often a temporary diagnostic designation. A fulminant disease course is common in unclassified colitis. Patients with unclassified colitis are more likely to require surgical interventions, and their complication rates are higher compared to those with Crohn’s disease or ulcerative colitis. Inflammatory bowel disease may also present with symptoms outside the gastrointestinal tract, known as extraintestinal manifestations. Extraintestinal manifestations of inflammatory bowel disease may include arthropathies, skin lesions, ocular involvement, and hepatopathies [11]. Due to the complex disease course, the number of hospitalizations may increase. Research has shown that patients with inflammatory bowel disease experience elevated stress levels due to the challenges of managing the condition [12]. Therefore, we have offered complementary medical consultations for patients with IBD via the physician-moderated online platform (https://www.ced-hospital.de/, accessed on 6 January 2003). In a secure, closed forum, this service allows patients to ask questions and stay in touch beyond the usual clinic visits. Analysis of forum interactions can uncover persistent information gaps after traditional outpatient consultations and provide valuable insights into patient needs and concerns. These insights can lead to improvements in patient education, communication strategies, and overall care processes for the benefit of both patients and healthcare providers. Considering the numerous challenges in disease management and the high socioeconomic burden of inflammatory bowel disease, we aimed to investigate patient inquiries in our online consultation forum. The specific information gaps among patients with inflammatory bowel disease have not been thoroughly investigated. This is the focus of our study.

## 2. Material and Methods

### 2.1. Study Design and Data Source

This study is a retrospective monocenter cohort study of a closed internet forum (https://www.ced-hospital.de/) of patients with a chronic inflammatory bowel disease conducted at the Asklepios Westklinikum Hamburg, Germany. The Department of Internal Medicine and Gastroenterology is a tertiary care center specializing in the treatment of inflammatory bowel disease. Our data collection for this analysis started in January 2003 (founding year of the forum) and was repeated in 2013 and 2024, with each data collection spanning a 12-month period. We chose intervals of at least 10 years between each survey to effectively observe and analyze long-term changes.

### 2.2. Participants

Our inclusion and exclusion criteria were as follows:

Inclusion criteria:

- Diagnosis of a chronic inflammatory bowel disease;
- Adults aged 18 years and older;
- Capacity to give consent;
- Prior consultation with an internist in the outpatient clinic.

Exclusion criteria:

- Age under 18 years;
- Incapacity to give consent.

**Figure 1:**
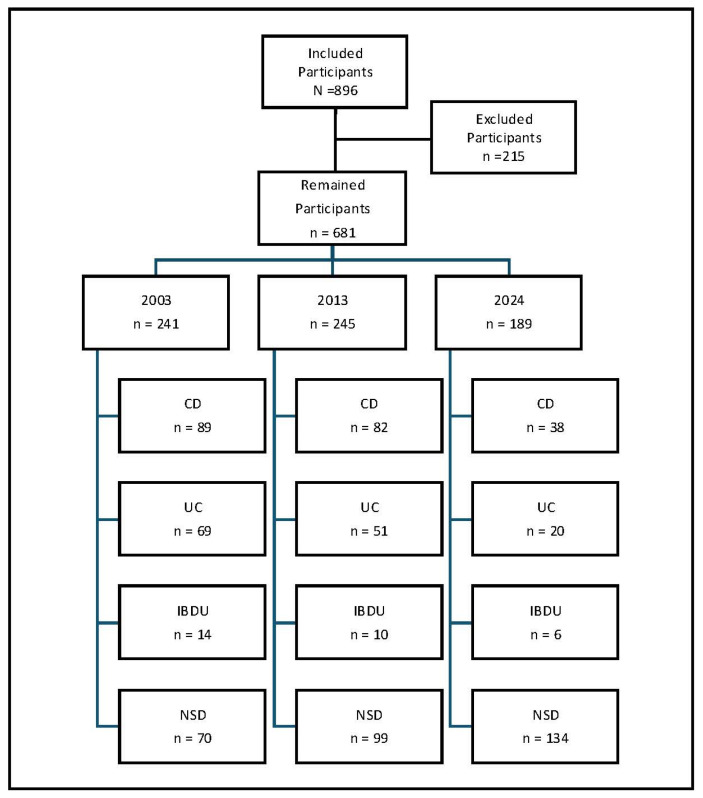
Flow chart of the study participants.

We did not consider contributions that contained a comment (e.g., thanks and greetings) or information (e.g., preferred appointment times) for the team. Contributions that had no relevance to the topic of chronic inflammatory bowel disease were excluded. After applying these criteria, 681 cases remained in the final dataset (Figure 1).

### 2.3. Ethical Statement

Permission for this study was obtained from the Ethics Committee of the Medical Faculty of the Christian-Albrechts University of Kiel, Ethics application no.: D 432/21.

### 2.4. Statistical Analysis

We categorized participants based on their clinical profiles. These categories included Crohn’s disease (CD), ulcerative colitis (UC), unclassified colitis (IBDU), and no specified diagnosis (NSD). We documented the content of each consultation and subsequently coded the information as binary variables (“present = 1” or “absent = 0”). We used SPSS for Windows, version 29.0 (SPSS Inc., Chicago, IL, USA), for data analysis. We selected the chi-square test and Fisher’s exact test as statistical methods. All statistical tests were two-sided, and a *p*-value of less than 0.01 was considered statistically significant. We set the significance level at 1% to minimize the probability of committing a Type I error because of alpha inflation (incorrectly rejecting the null hypothesis). This more stringent threshold ensures that only results with strong evidence are considered statistically significant, which is particularly important when the consequences of false positives are substantial.

## 3. Results

**Figure 2:**
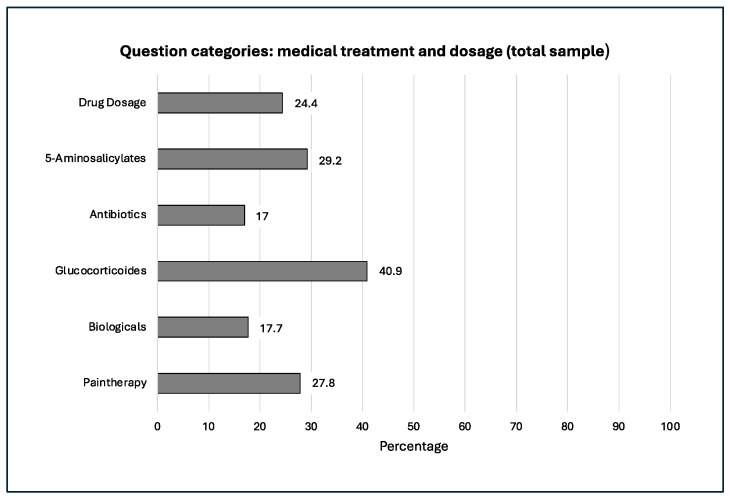
Therapy with glucocorticoids was discussed.

**Figure 3:**
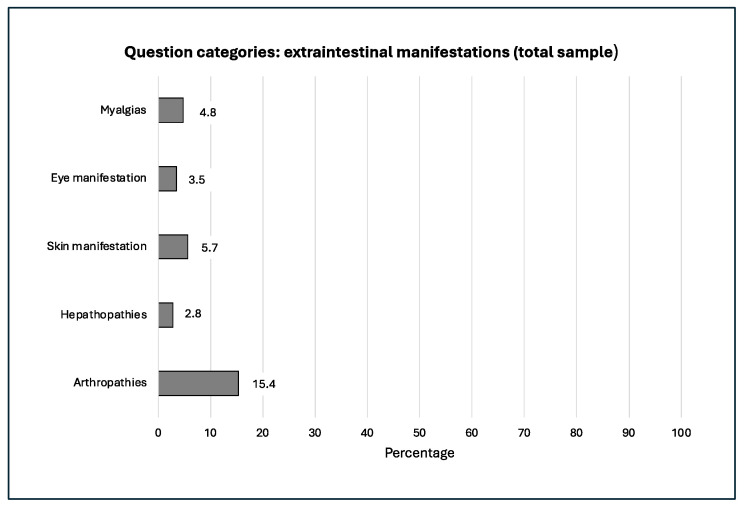
Arthropathies burdened.

**Figure 4:**
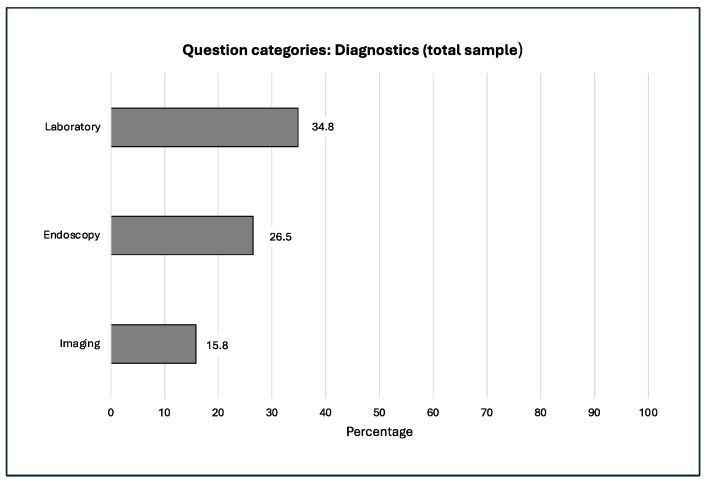
Laboratory tests were asked about.

**Figure 5:**
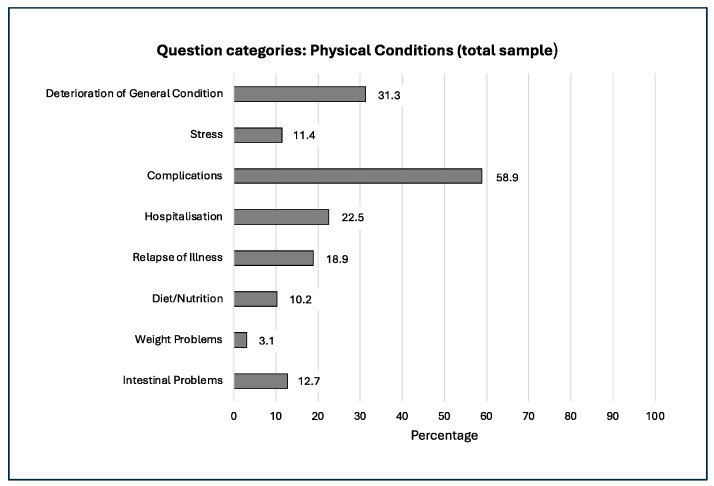
Complications caused concern.

**Figure 6:**
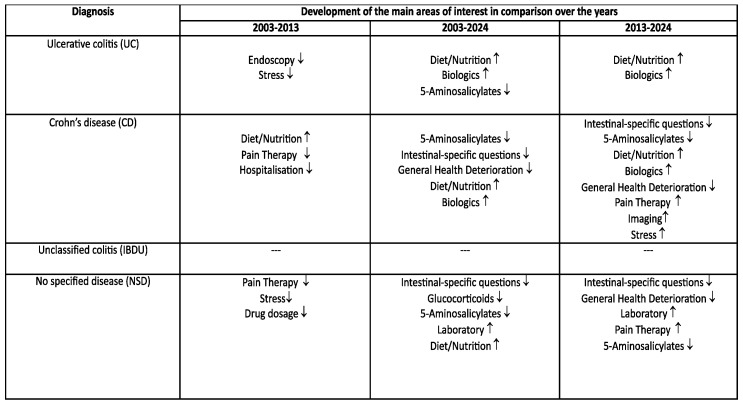
Over the years, there has been a shift in the focus of interest. Symbols: Increase in interest: ↑; decrease in interest: ↓.

The frequency distribution and progression of our question categories within our study over the 20 years examined is shown in the following Figure 2, Figure 3, Figure 4, Figure 5 and Figure 6.

The analysis of the consultation topics and focal points across the four categories (CD, UC, IB-DU, and NSD) showed significant changes over the years studied, namely 2003, 2013, and 2024. Comparisons between 2003 and 2013, 2003 and 2024, and 2013 and 2024 all revealed notable differences in the topics and focal points (Table 1, Table 2, Table 3 and Table 4).

### 3.1. Ulcerative Colitis (UC)

1. Comparing consultation topics between 2003 and 2013 revealed significant changes in patient interests, with notable declines in interest regarding endoscopy (*p* = 0.007) and stress (*p* = 0.001) over the years (Table 1).
2. Significant differences were observed when comparing 2003 and 2024: there was an increased interest in diet and nutritional management (*p* < 0.001) as well as in treatment with biologics (*p* < 0.001), while interest in 5-aminosalicylate treatment decreased (*p* < 0.001).
3. In comparing 2013 and 2024, there was a significant increase in diet/nutritional management (*p* = 0.004) and biologics (*p* < 0.001).

### 3.2. Crohn’s Disease (CD)

1. The comparison between 2003 and 2013 revealed a significant increase in interest in diet and nutritional management (*p* = 0.001), while interest in hospitalization (*p* = 0.007) and pain therapy (*p* = 0.002) significantly declined (Table 2).
2. When comparing 2003 and 2024, interest in bowel-specific problems (<0.001), 5-aminosalicylate treatment (*p* < 0.001), and general health deterioration (*p* = 0.003) decreased, while interest in diet and nutritional management (*p* < 0.001) and biologic drug therapy (*p* < 0.001) increased.
3. The comparison between 2013 and 2024 showed a decrease in interest regarding bowel-specific problems (*p* = 0.003), 5-aminosalicylate treatment (*p* < 0.001), and overall health deterioration (*p* < 0.001). Conversely, there was a significant increase in interest in diet and nutritional management (*p* = 0.002), pain therapy (*p* < 0.001), biologics (*p* < 0.001), diagnostic imaging procedures (*p* = 0.004), and stress management (*p* < 0.001).

### 3.3. Unclassified Colitis (IBDU)

No significant events occurred in this group when comparing the years (Table 3).

### 3.4. No Specified Disease (NSD)

1. There was a decline in interest regarding pain management (*p* = 0.002), medication dosage (*p* = 0.006), and stress reduction (*p* = 0.009) (Table 4).
2. Between 2003 and 2024, there was a significant decline in interest regarding the following topics: bowel-specific problems (*p* < 0.001), glucocorticoids (*p* = 0.004), and 5-aminosalicylates (*p* = 0.004). An increase was noted in laboratory diagnostics (*p* < 0.001) as well as in aspects related to diet and nutrition (*p* < 0.001).
3. A comparison between 2013 and 2024 again revealed significant differences. There was a decrease in interest regarding bowel-specific problems (*p* < 0.001), 5-aminosalicylate treatment (*p* < 0.001), and general health deterioration (*p* < 0.001). Conversely, interest increased in pain therapy (*p* < 0.001) and laboratory diagnostics (*p* < 0.001).

The results of the comparisons of question categories between years for each disease entity are summarized in the above tables.

## 4. Discussion

Our study revealed a shift in interests among forum participants over time. Patients with ulcerative colitis initially expressed interest in endoscopic examinations and stress management. Over time, this focus shifted towards topics such as diet and nutrition, as well as treatment with biologics. The interim interest in therapy with 5-aminosalicylates diminished in significance over the course of the study period. Patients with ulcerative colitis may experience recurrent disease exacerbations triggered by prolonged psychological stress, often necessitating endoscopic evaluations. This provides a plausible connection that aligns with the observed clinical interests [13,14]. Patients with Crohn’s disease also showed increasing interest in stress at the end of the study. Stressful situations can significantly impair the quality of life of patients with inflammatory bowel disease by intensifying their physical sensations and contributing to a range of psychosomatic problems [12,15]. In this context, it is likely that interest in the topic of stress has increased over time. Patients with Crohn’s disease also expressed concerns about the deterioration of their general health, although these concerns appeared to decrease over time. Similarly, individuals without a specific disease were concerned about a general deterioration in their health, which initially increased before gradually decreasing. In addition to stress, pain management was also addressed, as pain itself could serve as a stressor. Patients with Crohn’s disease initially showed interest in pain therapy, which decreased and then increased again over time. The same phenomenon was observed in patients with no specified disease. Stress played no further role in the patients with no specified disease during the study. Abdominal pain was a common trigger for discomfort in patients with inflammatory bowel disease and can impair their quality of life. Up to 60% of patients with Crohn’s disease or ulcerative colitis suffer from chronic pain, which can lead to significant psychological stress [16]. According to the literature, patients with inflammatory bowel disease often experience pain beyond the intestines, with common locations including the back, joints, and head. Research suggests that pain tends to be more persistent in patients with Crohn’s disease compared to those with ulcerative colitis, highlighting potential differences in disease management and treatment outcomes [17,18]. The heightened pain symptoms experienced by patients with Crohn’s disease may contribute to their ongoing interest in pain management. This is likely due to the fact that effective pain relief is crucial for improving their quality of life. Patients with ulcerative colitis have shown interest in therapy with biologics. The introduction of biologics, particularly anti-TNF-alpha, in 2003 may have contributed to this trend. Treatment with biologics can lead to more rapid modulation of inflammatory processes, potentially resulting in longer symptom-free intervals for patients [19,20]. An additional explanation for this trend is also the risk of developing pouchitis in patients with ulcerative colitis. Pouchitis frequently fails to respond adequately to conventional antibiotic treatment. The introduction of biologics such as vedolizumab in 2014 has provided a promising new therapeutic option. Given the encouraging clinical outcomes associated with vedolizumab in the management of pouchitis, this may have contributed to the heightened interest in biologic treatments among patients with ulcerative colitis [21]. Patients with ulcerative colitis showed increased interest in diet and nutrition in 2024 compared to previous years. From 2013 onwards, diet and nutrition also played a significant role for patients with Crohn’s disease, and this interest continued throughout the study. Individuals with no specified disease also demonstrated increased interest in nutrition, which became an important topic in 2024. In patients with ulcerative colitis, there is evidence that a good nutritional status appears to influence disease activity, symptom severity, and the risk of undergoing surgery [22]. One potential explanation for the increased interest is the emerging evidence suggesting that diet may influence the pathogenesis of inflammatory bowel disease, and that dietary and nutritional interventions could play a beneficial role in its management. Consequently, nutritional management is now recognized as an essential component of inflammatory bowel disease treatment, and guidelines have been established to support this approach [23]. As a result, preventive nutrition is increasingly being recommended as a complement to traditional nutritional management, particularly among patients with Crohn’s disease [24]. Our study highlights the importance of nutrition in inflammatory bowel disease care in patients’ everyday lives. Crohn’s disease patients and individuals with no specified disease showed interest in obtaining information about treatment with 5-aminosalicates. This interest significantly declined as the study progressed. Among patients with no specified disease, the interest in 5-aminosalicylates also decreased over time. Despite their limited efficacy in the treatment in inflammatory bowel disease, especially of Crohn’s disease, 5-aminosalicylates continue to be widely used, even though they are not recommended for either induction therapy or maintenance of remission. Their ongoing use despite the lack of robust supporting evidence may help explain the sustained interest in this treatment option [25,26]. During an inflammatory bowel disease episode, patients with chronic inflammatory bowel disease may experience malabsorption or malnutrition, leading to symptoms like diarrhea and abdominal pain [27]. The symptoms can occur at any time, even during periods of remission. Fortunately, advances in treatment options have improved symptom management, potentially reducing the burden of these issues for patients with Crohn’s disease and patients with no specified disease over time. This is demonstrated by the decrease in expressed interest among patients with Crohn’s disease and those with no disease mentioned. Hospitalization represented a significant concern for patients with Crohn’s disease; however, its relative importance diminished over the course of the study. Research has shown that Crohn’s disease patients tend to experience more severe complications, resulting in higher hospitalization rates compared to those with ulcerative colitis. This may be due to the fact that Crohn’s disease is often characterized by severe disease progression and additional complications, such as fistulas or stenoses, which require more intensive medical care. As a result, patients with Crohn’s disease may have a greater interest in understanding and managing hospitalization-related topics and complications [28]. Laboratory diagnostics, in particular, gained popularity among individuals with no specified disease, with a notable increase in 2024. The use of fecal calprotectin as a diagnostic test has become more widespread in recent years, providing a reliable and non-invasive means of assessing disease activity. This has led to a reduction in the need for invasive colonoscopies, which in turn has decreased the physical burden on patients and may have contributed to an increase in interest in this topic [29]. Imaging techniques are becoming increasingly important in Crohn’s disease. Conventional imaging techniques such as computed tomography and magnetic resonance imaging (MRI) are being complemented by newer technologies such as capsule endoscopy. Since 2010, capsule endoscopy in Germany has been covered by statutory health insurance for unexplained minor bowel bleeding, especially in patients with Crohn’s disease. This technology has proven highly effective in detecting small intestine lesions in patients with Crohn’s disease. Additionally, MRI has emerged as a safe and reliable method for examining the small intestine. Imaging procedures provide a non-invasive alternative to endoscopic exams, allowing for the clarification of specific intestinal issues and offering significant benefits and relief to patients with inflammatory bowel disease [30]. In individuals with no specified disease, topics such as medication dosages and glucocorticoid therapy were initially of interest, although this interest diminished over time. The interest in general health deterioration may be attributed to the complexity of the topic [31]. The observed shifts in topics of interest over time may reflect the dynamic and evolving needs of individuals living with inflammatory bowel disease. These changes underscore the necessity of continuous monitoring and responsiveness to patient priorities in order to facilitate personalized treatment strategies and targeted counseling. Such an adaptive approach may not only enhance therapeutic outcomes but also help mitigate potential information deficits among patients. Our findings are subject to several limitations, including a small sample size. Another limitation is the absence of direct data collection through questionnaires, which could have provided more accurate and detailed insights. For instance, a questionnaire might have allowed for clearer distinctions between different types of pain. Further research is needed to assess the time and resources required to organize and manage online forums, an understanding that could help healthcare providers develop effective time management strategies. Despite these limitations, our study suggests that online counseling could serve as a valuable supplement to traditional counseling for patients with inflammatory bowel disease. While the small sample size restricts the generalizability of our findings, they nonetheless offer initial insights into the needs that may be relevant for supporting individual patients and their families. A follow-up study would be beneficial to validate these preliminary results.

## 5. Conclusions

Our study suggests that patient-centered care may be beneficial for individuals with inflammatory bowel disease. The diverse and changing needs and interests of patients with inflammatory bowel disease highlight the potential value of personalized therapeutic approaches that consider the physical, psychological, and social aspects of the disease. Online counseling may offer an accessible way to support patients with inflammatory bowel disease, and our findings could inform the development of tailored interventions and guidelines for healthcare providers. An online platform can be used to identify information gaps in patients with inflammatory bowel disease and to address potential uncertainties related to their therapy. Further research is needed to explore the long-term effects of different therapies, investigate the role of nutrition and lifestyle in inflammatory bowel disease management, and develop digital health resources that support patients and their families. By exploring these avenues, we may be able to improve the quality of life for people with inflammatory bowel disease and promote better health outcomes.

## Figures and Tables

**Table 1:** Comparison of topics of interest among patients with ulcerative colitis.

Ulcerative Colitis (UC)									
	Years and *p*-Values			Years and *p*-Values			Years and *p*-Values		
Question Categories	2003	2013	*p*	2003	2024	*p*	2013	2024	*p*
Intestinal-specific questions	7.4% (n = 5)	21.6% (n = 11)	0.031	7.4% (n = 5)	0.0% (n = 0)	0.584	21.6% (n = 11)	0.0% (n = 0)	0.027
Weight problems	1.5% (n = 1)	2.0% (n = 1)	1.000	1.5% (n = 1)	15.0% (n = 3)	0.035	2.0% (n = 1)	15.0% (n = 3)	0.065
Diet/nutrition	2.9% (n = 2)	5.9% (n = 3)	0.650	2.9% (n = 2)	35.0% (n = 7)	**<0.001**	5.9% (n = 3)	35.0% (n = 7)	**0.004**
Disease relapse	47.1% (n = 32)	27.5% (n = 14)	0.037	47.1% (n = 32)	40.0% (n = 8)	0.619	27.5% (n = 14)	40.0% (n = 8)	0.394
Hospitalization	20.6% (n = 14)	27.5% (n = 14)	0.393	20.6% (n = 14)	25.0% (n = 5)	0.759	27.5% (n = 14)	25.0% (n = 5)	1.00
Complications	75.0% (n = 51)	56.9% (n = 29)	0.049	75.0% (n = 51)	70.0% (n = 14)	0.773	56.9% (n = 29)	70.0% (n = 14)	0.420
Arthropathies	23.5% (n = 16)	15.7% (n = 8)	0.359	23.5% (n = 16)	20.0% (n = 4)	1.000	15.7% (n = 8)	20.0% (n = 4)	0.729
Hepatopathies	1.5% (n = 1)	5.9% (n = 3)	0.312	1.5% (n = 1)	0.0% (n = 0)	1.000	5.9% (n = 3)	0.0% (n = 0)	0.554
Skin-related disease manifestations	0.0% (n = 0)	9.8% (n = 5)	0.013	0.0% (n = 0)	0.0% (n = 0)	1.000	9.8% (n = 5)	0.0% (n = 0)	0.312
Eye-related disease manifestations	4.4% (n = 3)	7.8% (n = 4)	0.460	4.4% (n = 3)	0.0% (n = 0)	1.000	7.8% (n = 4)	0.0% (n = 0)	0.571
Myalgia	0.0% (n = 0)	3.9% (n = 2)	0.182	0.0% (n = 0)	10.0% (n = 2)	0.050	3.9% (n = 2)	10.0% (n = 2)	0.314
Pain therapy	38.2% (n = 26)	23.5% (n = 12)	0.113	38.2% (n = 26)	55.0% (n = 11)	0.206	23.5% (n = 12)	11.0% (n = 11)	0.022
Biologics	1.5% (n = 1)	5.9% (n = 3)	0.312	1.5% (n = 1)	50.0% (n = 10)	**<0.001**	5.9% (n = 3)	50.0% (n = 10)	**<0.001**
Glucocorticoids	61.8% (n = 42)	41.2% (n = 21)	0.041	61.8% (n = 42)	40.0% (n = 8)	0.123	41.2% (n = 21)	40.0% (n = 8)	1.000
Antibiotics	22.1% (n = 15)	23.5% (n = 12)	1.000	22.1% (n = 15)	10.0% (n = 2)	0.339	23.5% (n = 12)	10.0% (n = 2)	0.321
5-Aminosalicylates	75.0% (n = 51)	51.0% (n = 26)	0.011	75.0% (n = 51)	20.0% (n = 4)	**<0.001**	51.0% (n = 26)	20.0% (n = 4)	0.031
Imaging	11.8% (n = 8)	11.8% (n = 6)	1.000	11.8% (n = 8)	25.0% (n = 5)	0.161	11.8% (n = 6)	25.0% (n = 5)	0.272
Endoscopy	47.1% (n = 32)	21.6% (n = 11)	**0.007**	47.1% (n = 32)	20.0% (n = 4)	0.039	21.6% (n = 11)	20.0% (n = 4)	1.000
Laboratory	26.5% (n = 18)	39.2% (n = 20)	0.166	26.5% (n = 18)	35.0% (n = 7)	0.574	39.2% (n = 20)	35.0% (n = 7)	0.792
Drug dosage	32.4% (n = 22)	27.5% (n = 14)	0.687	32.4% (n = 22)	25.0% (n = 5)	0.594	27.5% (n = 14)	25.0% (n = 5)	1.000
Stress	26.5% (n = 18)	3.9% (n = 2)	**0.001**	26.5% (n = 18)	15.0% (n = 3)	0.380	3.9% (n = 2)	15.0% (n = 3)	0.132
General health deterioration	39.7% (n = 27)	47.1% (n = 24)	0.458	39.7% (n = 27)	35.0% (n = 7)	0.797	47.1% (n = 24)	35.0% (n = 7)	0.431

**Table 2:** Comparison of topics of interest among patients with Crohn’s disease.

Crohn’s Disease (CD)									
	Years and *p*-Values			Years and *p*-Values			Years and *p*-Values		
Question Categories	2003	2013	*p*	2003	2024	*p*	2013	2024	*p*
Intestinal-specific questions	22.5% (n = 20)	19.5% (n = 16)	0.709	22.5% (n = 20)	0.0% (n = 0)	**<0.001**	19.5% (n = 16)	0.0% (n = 0)	**0.003**
Weight problems	1.1% (n = 1)	7.3% (n = 6)	0.056	1.1% (n = 1)	0.0% (n = 0)	1.000	7.3% (n = 6)	0.0% (n = 0)	0.175
Diet/nutrition	0.0% (n = 0)	11.0% (n = 9)	**0.001**	0.0% (n = 0)	36.8% (n = 14)	**<0.001**	11.0% (n = 9)	36.8% (n = 14)	**0.002**
Disease relapse	23.6% (n = 21)	17.1% (n = 14)	0.345	23.6% (n = 21)	26.3% (n = 10)	0.822	17.1% (n = 14)	26.3% (n = 10)	0.326
Hospitalization	37.1% (n = 33)	18.3% (n = 15)	**0.007**	37.1% (n = 33)	31.6% (n = 12)	0.686	18.3% (n = 15)	31.6% (n = 12)	0.157
Complications	65.2% (n = 58)	61.0% (n = 50)	0.635	65.2% (n = 58)	52.6% (n = 20)	0.233	61.0% (n = 50)	52.6% (n = 20)	0.430
Arthropathies	22.5% (n = 20)	20.7% (n = 17)	0.853	22.5% (n = 20)	18.4% (n = 7)	0.813	20.7% (n = 17)	18.4% (n = 7)	1.000
Hepatopathies	2.2% (n = 2)	7.3% (n = 6)	0.155	2.2% (n = 2)	0.0% (n = 0)	1.000	7.3% (n = 6)	0.0% (n = 0)	0.175
Skin-related disease manifestations	3.4% (n = 3)	14.6% (n = 12)	0.013	3.4% (n = 3)	7.9% (n = 3)	0.363	14.6% (n = 12)	7.9% (n = 3)	0.383
Eye-related disease manifestations	5.6% (n = 5)	7.3% (n = 6)	0.760	5.6% (n = 5)	5.3% (n = 2)	1.000	7.3% (n = 6)	5.3% (n = 2)	1.000
Myalgia	1.1% (n = 1)	9.8% (n = 8)	0.015	1.1% (n = 1)	5.3% (n = 2)	0.213	9.8% (n = 8)	5.3% (n = 2)	0.501
Pain therapy	32.6% (n = 29)	12.2% (n = 10)	**0.002**	32.6% (n = 29)	50.0% (n = 19)	0.074	12.2% (n = 10)	50.0% (n = 19)	**<0.001**
Biologics	18.0% (n = 16)	18.3% (n = 15)	1.000	18.0% (n = 16)	52.6% (n = 20)	**<0.001**	18.3% (n = 15)	52.6% (n = 20)	**<0.001**
Glucocorticoids	62.9% (n = 56)	45.1% (n = 37)	0.022	62.9% (n = 56)	50.0% (n = 19)	0.237	45.1% (n = 37)	50.0% (n = 19)	0.695
Antibiotics	19.1% (n = 17)	24.4% (n = 20)	0.459	19.1% (n = 17)	26.3% (n = 10)	0.356	24.4% (n = 20)	26.3% (n = 10)	0.824
5-Aminosalicylates	39.3% (n = 35)	34.1% (n = 28)	0.528	39.3% (n = 35)	0.0% (n = 0)	**<0.001**	34.1% (n = 28)	0.0% (n = 0)	**<0.001**
Imaging	19.1% (n = 17)	14.6% (n = 12)	0.542	19.1% (n = 17)	39.5% (n = 15)	0.025	14.6% (n = 12)	39.5% (n = 15)	**0.004**
Endoscopy	24.7% (n = 22)	22.0% (n = 18)	0.720	24.7% (n = 22)	34.2% (n = 13)	0.286	22.0% (n = 18)	34.2% (n = 13)	0.181
Laboratory	34.8% (n = 31)	32.9% (n = 27)	0.872	34.8% (n = 31)	52.6% (n = 20)	0.076	32.9% (n = 27)	52.6% (n = 20)	0.046
Drug dosage	28.1% (n = 25)	15.9% (n = 13)	0.066	28.1% (n = 25)	36.8% (n = 14)	0.401	15.9% (n = 13)	36.8% (n = 14)	0.018
Stress	14.6% (n = 13)	3.7% (n = 3)	0.017	14.6% (n = 13)	26.3% (n = 10)	0.135	3.7% (n = 3)	26.3% (n = 10)	**<0.001**
General health deterioration	39.3% (n = 35)	48.8% (n = 40)	0.222	39.3% (n = 35)	13.2% (n = 5)	**0.003**	48.8% (n = 40)	13.2% (n = 5)	**<0.001**

**Table 3:** Comparison of topics of interest among patients with unclassified colitis.

Unclassified Colitis (IBDU)									
	Years and *p*-Values			Years and *p*-Values			Years and *p*-Values		
Question Categories	2003	2013	*p*	2003	2024	*p*	2013	2024	*p*
Intestinal-specific questions	42.9% (n = 6)	40.0% (n = 4)	1.000	42.9% (n = 6)	0.0% (n = 0)	0.115	40.0% (n = 4)	0.0% (n = 0)	0.234
Weight problems	0.0% (n = 0)	10% (n = 1)	0.417	0.0% (n = 0)	33.3% (n = 2)	0.079	10.0% (n = 1)	33.3% (n = 2)	0.518
Diet/nutrition	0.0% (n = 0)	30.0% (n = 3)	0.059	0.0% (n = 0)	33.3% (n = 2)	0.079	30.0% (n = 3)	33.3% (n = 2)	1.000
Disease relapse	7.1% (n = 1)	50.0% (n = 5)	0.050	7.1% (n = 1)	33.3% (n = 2)	0.202	50.0% (n = 5)	33.3% (n = 2)	0.633
Hospitalization	35.7% (n = 5)	60.0% (n = 6)	0.408	35.7% (n = 5)	16.7% (n = 1)	0.613	60.0% (n = 6)	16.7% (n = 1)	0.145
Complications	78.6% (n = 11)	80.0% (n = 8)	1.000	78.6% (n = 11)	50.0% (n = 3)	0.303	80.0% (n = 8)	50.0% (n = 3)	0.299
Arthropathies	14.3% (n = 2)	30.0% (n = 3)	0.615	14.3% (n = 2)	16.7% (n = 1)	1.000	30.0% (n = 3)	16.7% (n = 1)	1.000
Hepatopathies	0.0% (n = 0)	30.0% (n = 3)	0.059	0.0% (n = 0)	0.0% (n = 0)	1.000	30.0% (n = 3)	0.0% (n = 0)	0.250
Skin-related disease manifestations	0.0% (n = 0)	20.0% (n = 2)	0.163	0.0% (n = 0)	0.0% (n = 0)	1.000	20.0% (n = 2)	0.0% (n = 0)	0.500
Eye-related disease manifestations	0.0% (n = 0)	20.0% (n = 2)	0.163	0.0% (n = 0)	16.7% (n = 1)	0.300	20.0% (n = 2)	16.7% (n = 1)	1.000
Myalgia	0.0% (n = 0)	30.0% (n = 3)	0.059	0.0% (n = 0)	16.7% (n = 1)	0.300	30.0% (n = 3)	16.7% (n = 1)	1.000
Pain therapy	35.7% (n = 5)	50.0% (n = 5)	0.678	35.7% (n = 5)	66.7% (n = 4)	0.336	50.0% (n = 5)	66.7% (n = 4)	0.633
Biologics	14.3% (n = 2)	10.0% (n = 1)	1.000	14.3% (n = 2)	66.7% (n = 4)	0.037	10.0% (n = 1)	66.7% (n = 4)	0.036
Glucocorticoids	42.9% (n = 6)	50.0% (n = 5)	1.000	42.9% (n = 6)	50.0% (n = 3)	1.000	50.0% (n = 5)	50.0% (n = 3)	1.000
Antibiotics	21.4% (n = 3)	40.0% (n = 4)	0.393	21.4% (n = 3)	50.0% (n = 3)	0.303	40.0% (n = 4)	50.0% (n = 3)	1.000
5-Aminosalicylates	35.7% (n = 5)	30.0% (n = 3)	1.000	35.7% (n = 5)	16.7% (n = 1)	0.613	30.0% (n = 3)	16.7% (n = 1)	1.000
Imaging	14.3% (n = 14)	40.0% (n = 10)	0.192	14.3% (n = 2)	50.0% (n = 3)	0.131	40.0% (n = 4)	50.0% (n = 3)	1.000
Endoscopy	50.0% (n = 7)	70.0% (n = 7)	0.421	50.0% (n = 7)	50.0% (n = 3)	1.000	70.0% (n = 7)	50.0% (n = 3)	0.607
Laboratory	35.7% (n = 5)	70.0% (n = 7)	0.214	35.7% (n = 5)	66.7% (n = 4)	0.336	70.0% (n = 7)	66.7% (n = 4)	1.000
Drug dosage	35.7% (n = 5)	70.0% (n = 7)	0.214	35.7% (n = 5)	50.0% (n = 3)	0.642	70.0% (n = 7)	50.0% (n = 3)	0.607
Stress	21.4% (n = 3)	20.0% (n = 2)	1.000	21.4% (n = 3)	16.7% (n = 1)	1.000	20.0% (n = 2)	16.7% (n = 1)	1.000
General health deterioration	50.0% (n = 7)	60.0% (n = 6)	0.697	50.0% (n = 7)	33.3% (n = 2)	0.642	60.0% (n = 6)	33.3% (n = 2)	0.608

**Table 4:** Comparison of topics of interest among patients with no specified disease.

No Specified Disease (NSD)									
	Years and *p*-Values			Years and *p*-Values			Years and *p*-Values		
Question Categories	2003	2013	*p*	2003	2024	*p*	2013	2024	*p*
Intestinal-specific questions	14.3% (n = 10)	14.1% (n = 14)	1.000	14.3% (n = 10)	0.0% (n = 0)	**<0.001**	14.1% (n = 14)	0.0% (n = 0)	**<0.001**
Weight problems	1.4% (n = 1)	2.0% (n = 2)	1.000	1.4% (n = 1)	2.2% (n = 3)	1.000	2.0% (n = 2)	2.2% (n = 3)	1.000
Diet/nutrition	1.4% (n = 1)	6.1% (n = 6)	0.241	1.4% (n = 1)	17.2% (n = 23)	**<0.001**	6.1% (n = 6)	17.2% (n = 23)	0.015
Disease relapse	7.1% (n = 5)	7.1% (n = 7)	1.000	7.1% (n = 5)	6.0% (n = 8)	0.768	7.1% (n = 7)	6.0% (n = 8)	0.791
Hospitalization	17.1% (n = 12)	20.2% (n = 20)	0.693	17.1% (n = 12)	11.9% (n = 16)	0.391	20.2% (n = 20)	11.9% (n = 16)	0.100
Complications	47.1% (n = 33)	60.6% (n = 60)	0.087	47.1% (n = 33)	47.8% (n = 64)	1.000	60.6% (n = 60)	47.8% (n = 64)	0.063
Arthropathies	7.1% (n = 5)	9.1% (n = 9)	0.780	7.1% (n = 5)	9.0% (n = 12)	0.793	9.1% (n = 9)	9.0% (n = 12)	1.000
Hepatopathies	1.4% (n = 1)	2.0% (n = 2)	1.000	1.4% (n = 1)	0.0% (n = 0)	0.343	2.0% (n = 2)	0.0% (n = 0)	0.179
Skin-related disease manifestations	0.0% (n = 0)	8.1% (n = 8)	0.021	0.0% (n = 0)	3.7% (n = 5)	0.167	8.1% (n = 8)	3.7% (n = 5)	0.163
Eye-related disease manifestations	0.0% (n = 0)	0.7% (n = 1)	1.000	0.0% (n = 0)	0.7% (n = 1)	1.000	0.0% (n = 0)	0.7% (n = 1)	1.000
Myalgia	0.0% (n = 0)	3.0% (n = 3)	0.268	0.0% (n = 0)	8.2% (n = 11)	0.017	3.0% (n = 3)	8.2% (n = 11)	0.161
Pain therapy	25.7% (n = 18)	8.1% (n = 8)	**0.002**	25.7% (n = 18)	32.1% (n = 43)	0.421	8.1% (n = 8)	32.1% (n = 43)	**<0.001**
Biologics	12.9% (n = 9)	12.1% (n = 12)	1.000	12.9% (n = 9)	20.1% (n = 27)	0.247	12.1% (n = 12)	20.1% (n = 27)	0.114
Glucocorticoids	38.6% (n = 27)	29.3% (n = 29)	0.246	38.6% (n = 27)	18.7% (n = 25)	**0.004**	29.3% (n = 29)	18.7% (n = 25)	0.061
Antibiotics	4.3% (n = 3)	10.1% (n = 10)	0.242	4.3% (n = 3)	11.9% (n = 16)	0.082	10.1% (n = 10)	11.9% (n = 16)	0.834
5-Aminosalicylates	20.0% (n = 14)	25.3% (n = 25)	0.463	20.0% (n = 14)	6.0% (n = 8)	**0.004**	25.3% (n = 25)	6,0% (n = 8)	**<0.001**
Imaging	8.6% (n = 6)	10.1% (n = 10)	0.796	8.6% (n = 6)	14.9% (n = 20)	0.269	10.1% (n = 10)	14.9% (n = 20)	0.326
Endoscopy	18.6% (n = 13)	23.2% (n = 23)	0.568	18.6% (n = 13)	20.1% (n = 27)	0.854	23.2% (n = 23)	20.1% (n = 27)	0.629
Laboratory	17.1% (n = 12)	17.2% (n = 17)	1.000	17.1% (n = 12)	51.5% (n = 69)	**<0.001**	17.2% (n = 17)	51.5% (n = 69)	**<0.001**
Drug dosage	31.4% (n = 22)	13.1% (n = 13)	**0.006**	31.4% (n = 22)	17.2% (n = 23)	0.032	13.1% (n = 13)	17.2% (n = 23)	0.465
Stress	14.3% (n = 10)	3.0% (n = 3)	**0.009**	14.3% (n = 10)	7.5% (n = 10)	0.140	3.0% (n = 3)	7.5% (n = 10)	0.247
General health deterioration	14.3% (n = 10)	32.3% (n = 32)	0.011	14.3% (n = 10)	13.4% (n = 18)	0.834	32.3% (n = 32)	13.4% (n = 18)	**<0.001**

## Data Availability

The data underlying this article cannot be shared publicly due to the privacy of individuals who participated in the study. The data will be shared on reasonable request to the corresponding author.
